# Biological equivalence of additive manufacturing titanium vs conventional implants in peri-implantitis

**DOI:** 10.1590/0103-644020256596

**Published:** 2026-01-30

**Authors:** Pedro Henrique de Azambuja Carvalho, Ana Paula Farnezi Bassi, Vinícius Ferreira Bizelli, Julia Ferrazoli de Oliveira, Marisa Aparecida Cabrini Gabrielli, Valfrido Antonio Pereira-Filho

**Affiliations:** 1 Department of Diagnosis and Surgery, Division of Oral and Maxillofacial Surgery, School of Dentistry, São Paulo State University (UNESP), Araraquara, SP, Brazil; 2 Department of Diagnosis and Surgery, School of Dentistry, São Paulo State University (UNESP), Araçatuba, SP, Brazil

**Keywords:** peri-implantitis, titanium implants, additive manufacturing, inflammation

## Abstract

O aumento global benéfico no uso de implantes dentários também promove um aumento nas complicações relacionadas ao tratamento. A peri-implantite é uma questão preocupante nesse cenário. Este estudo teve como objetivo comparar o comportamento de um implante produzido por manufatura aditiva com implantes comercialmente disponíveis com diferentes tratamentos de superfície, expostos ou não á indução de peri-implantite. Oitenta implantes com três superfícies diferentes (Straumann BLT [A], Nobel Biocare Active [B], implante confeccionado em impressora 3D Plenum [C] e implante confeccionado em impressora 3D Plenum - usado como controle negativo [D]) foram colocados nas mandíbulas, em ambos os lados, de miniporcos machos (Minipig Br1). Três meses após a instalação dos implantes, a peri-implantite foi induzida com ligaduras de algodão em um dos lados da mandíbula, e um implante intraósseo serviu como controle em ambos os lados. A análise histométrica da perda óssea marginal não apresentou diferença estatística entre os grupos (p>0,05), mas em relação ao Contato Osso-Implante (BIC), o grupo B apresentou a maior porcentagem. O implante produzido por manufatura aditiva teve desempenho semelhante ao dos implantes comercialmente disponíveis em todas as análises realizadas, sugerindo que a manufatura aditiva pode ser uma alternativa para a fabricação de implantes osseointegrados.

## Abstract (en)

The global increase in the use of dental implants also increases treatment-related complications. Peri-implantitis is a matter of concern at this stage. This study aimed to compare the behavior of an additive manufacturing implant with commercially available implants with different surface treatments, exposed or not to a peri-implantitis challenge. Eighty implants with three different surfaces (Straumann BLT [A], Noble Biocare Active [B], Plenum 3D printed titanium implants test [C], and Plenum 3D printed titanium implants served as a negative control [D]) were placed into male minipigs (Minipig Br1) mandibles on both sides. Three months after implant placement, peri-implantitis was induced with cotton ligatures in one side of the mandible, and an intraosseous implant served as a control on both sides. Histometric analysis of marginal bone loss did not present a statistical difference between groups (p>0,05), but regarding Bone to Implant Contact (BIC), the B group presented the highest percentage. The additive manufacturing implant performed like commercially available implants in all the performed analyses, suggesting that additive manufacturing could be an alternative for the fabrication of osseointegrated implants.

**Figure:**
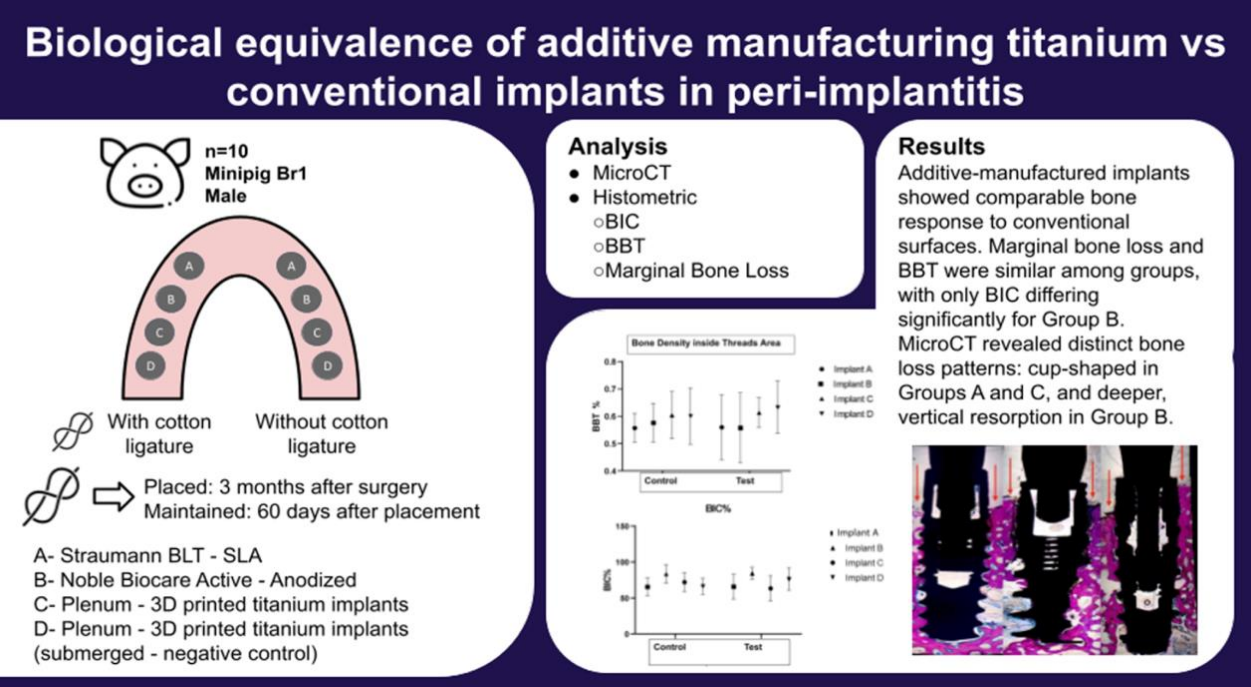

## Introduction

The use of dental implants to rehabilitate partial and edentulous subjects is a successful treatment in contemporary Dentistry. Overall, the global market of dental implants was valued at US$3 billion, with more than 9 million implants placed^1^. However, the prevalence of biological and technical complications regarding dental implant-supported restorations is growing at the same rate. Recent systematic reviews showed a primary concern about this scenario, wherein the prevalence of peri-implant diseases ranged between 0.4 to 43% after five years^2^ ^,^ ^3^

The Consensus Report of workgroup 4 of the 2017 World Workshop on the Classification of Periodontal and Peri-Implant Diseases and Conditions^4^ stated that peri-implantitis is an inflammatory process from a microbial origin that causes bone loss and, if not treated, could lead to the loss of the implant-supported restoration. Peri-implantitis is induced by a polymicrobial biofilm^4^ ^,^ ^5^, while systemic and local factors may modulate its onset and progression^6^ ^,^ ^7^. However, bacterial in origin, factors such as biomechanical overload, systemic bone metabolism disorders such as osteoporosis, and surgical or anatomical characteristics of the peri-implant site have been proposed as potential contributing factors^4^ ^,^ ^5^ ^,^ ^6^ ^,^ ^7^. 

Rougher surfaces have been associated with enhanced bone-to-implant contact and higher long-term clinical survival rates, highlighting the pivotal role of macro- and microgeometry in osseointegration^6^ ^,^ ^8^. Accordingly, over the past decade, several strategies have been developed to optimize implant design, focusing on surface modifications to increase roughness. These include titanium anodization, discrete calcium-phosphate crystal deposition, coatings with biological molecules, chemical modifications such as sandblasting, acid-etching, grit-blasting, and, more recently, implants produced by additive manufacturing, also referred to as 3D-printed surfaces^1^ ^,^ ^8^ ^,^ ^9^ ^,^ ^10^ ^,^ ^11^.

3D printing has been an expanding technology for dental implant surfaces due to its microstructure reproducibility, which can fulfill the demand for precision dental medicine^12^. In addition, emerging investigations demonstrate the reliability and predictability of implants with 3D-printed surfaces for numerous biological scenarios to stimulate the osseointegration^9^ ^,^ ^13^ ^,^ ^14^.

Nevertheless, considering the polymicrobial biofilm as a critical etiological factor of peri-implantitis, bacterial adhesion to implants represents a multifactorial process directly influenced by surface morphometry, including porosity and roughness^8^. Evidence also suggests that increased roughness may promote biofilm adhesion and, consequently, exacerbate peri-implant tissue destruction^6^ ^,^ ^7^ ^,^ ^8^. However, clinical findings remain inconclusive, as long-term studies have reported no significant differences in subgingival microbiota composition or peri-implantitis incidence between distinct implant surfaces^6^ ^,^ ^7^.

This duality underscores the ongoing challenge of promoting efficient osseointegration while minimizing susceptibility to bacterial colonization. Therefore, this animal study evaluated the behavior of an additive manufacturing implant with commercially available implants with different surface treatments when exposed to peri-implantitis challenge.

### Materials and methods

### Animals and Anesthesia

The study protocol was submitted to and approved by the Ethics Committee of the Albert Einstein Hospital Animal Facilities (CEUA #31832017) following the guidelines established by the Brazilian National Council for Animal Experimentation Control (CONSEA), using a study design that has been utilized in previous studies^9^ ^,^ ^15^ ^,^ ^16^.

Ten male minipigs (Minipig Br1), systemically healthy, were used; the sample size was defined based on the study by Shibli et al. (2003)^16^. The bone-to-implant contact (BIC) in the cervical third was used as the primary variable. A standard deviation of 7.3% and a minimum detectable difference of 13.4% were assumed. Considering a significance level of 5% (p=0.05) and a study power of 80%, the sample size was estimated at eight animals, with an additional 20% to account for potential loss, resulting in a total of 10 animals.

The animals were one-year-old with an average weight of 25 kg. All surgical and clinical procedures were performed under general anesthesia. The animals were anesthetized by intramuscular administration of ketamine hydrochloride (10mg/Kg) and midazolam (0.25mg/Kg). Propofol (5mg/Kg) was administered while using a funnel mask to induce anesthesia, which was maintained with 2% isoflurane by endotracheal intubation. Intraoperative analgesia was performed with Remifentanil (4ug/Kg/h) and postoperative analgesia with tramadol 2mg/Kg plus dipyrone 25mg/Kg TID for five days.

### Tooth Extraction

The extraction of all mandibular premolars and first molars created an edentulous ridge. The alveoli were allowed to heal for a period of 3 months. The upper premolars were also extracted to prevent occlusion trauma interference during plaque-induced peri-implantitis. Scaling and root planning were performed once a month until cotton ligatures were placed.

### Dental Implants, surface design, and surgery

A total of 80 dental implants with 3 different surfaces were used: 20 Nobel Biocare Active Internal RP implants with the anodized surface, 20 Straumann BLT Roxolid RB implants with SLA surface, 20 Plenum - 3D printed titanium implants, and 20 Plenum - 3D printed titanium implants- installed in submerged position on both sides of the mandible, which served as a negative control. The dental implants were installed on both sides of the mandible in the following order, from mesial to distal: Straumann (A), Nobel (B), Plenum (C), and Plenum - negative control (D). All Morse tapered implants were 10mm long and 3.5mm in diameter. Dental implants were placed 2mm in infra-crestal position after full-thickness flap surgery under aseptic conditions. The recipient sites were prepared according to the surgical techniques indicated by each implant manufacturer. After connecting the respective healing abutments (3.5 diameters and 4mm height), the flaps were sutured with single interrupted sutures. Sutures were removed after 15 days. The implants were distributed among the minipigs so that each dental implant surface could be represented on each mandibular side.

### Experimental peri-implantitis

After a healing period of 3 months after implant placement, cotton ligature wires were placed in a submarginal position around the dental implants and sutured in the peri-implant mucosa to hold the ligatures in position. The positions of the ligatures were checked monthly. Peri-implant bone loss was accelerated by tying different ligatures at 30-day intervals for 60 days.

**Figure 1:**
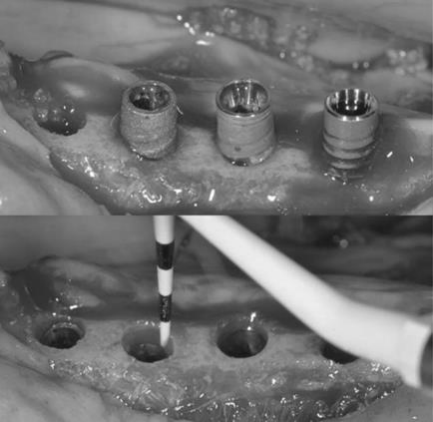
(A) Dental implants evaluated in the study; from right to left - Straumann, Nobel, Plenum, and the fourth site negative control - submerged Plenum; (B) Dental implant placement in infra-crestal position (2mm depth).

The allocation of the mandibular quadrants and a split-mouth design was established: on one side, dental implants without ligature, and on the other side, peri-implantitis induced para-cotton ligatures (Figure 1). The Plenum implant (group D) was placed on both sides in the molar area as a negative control implant (submerged implant, n=20 implants) at this stage of the experiment. Their purpose was to serve as baseline controls, allowing the assessment of the intrinsic osseointegration capacity of the additive manufacturing surface in the absence of bacterial or inflammatory challenge and enabling intra-animal comparison with the exposed implants^6^ ^,^ ^9^ ^,^ ^11^.

### Euthanasia and implant retrieval

Two months after dental plaque accumulation, the animals were sacrificed by induction of deep anesthesia followed by intravenous sodium pentobarbital euthanasia. The mandibles were remove d, and block biopsies of each implant site were dissected and fixed in 4% neutral formalin for 48 h.

### Micro-computed Tomography (microCT)

After histological fixation, the mandibles of minipigs were submitted to a MicroCT scan (Skyscan 1172, Aartselaar, Belgium) and NRecon software (version 1.6.10.4; SkyScan, Kontich, Belgium) plus CtAn (version 1.17.7.2; SkyScan) were used for visualization and scalar analysis of the samples. Using the mentioned software, the images acquired by the scanner were reconstructed to demonstrate 2D slices, and the files were three-dimensionally reconstructed in the DataViewer software (version 1.5.6.2; Bruker). The sample positions were determined by an experienced examiner, considering the dimensional irregularity of anatomical specimens.

### Histological procedures

The biopsies were then prepared for ground sectioning according to the previously described methods by Donath & Breuner (1982). First, the specimens were cut in a mesial-distal plane using a cutting-grinding unit (Exacts Cutting, System, Apparatebau Gmbh, Hamburg, Germany). One central section of each biopsy was prepared and reduced to a final thickness of 50-70 μm by micro-grinding and polishing using a micro-grinding unit (Exacts Cutting, System, Apparatebau GmbH). The sections were stained in toluidine blue to assess histometric parameters:^1^ bone loss - Distance from the bottom of the defect to the most coronal point of the newly formed bone with intimate contact with the implant surface,^2^ percentage of bone-to-implant contact (BIC - mineralized bone contact with the implant surface); and^3^ bone area within the limits of the implant threads at the portion of the implant (BBT - bone between threads)

### Data analysis

Statistical analyses were performed using the GraphPad Prism 5 program. After homogeneity and variance tests, non-parametric analysis was used to evaluate the histometric variables. Next, differences between groups and intra-group were assessed using Kruskal-Wallis with Dunn's post-test and Friedman's test with Dunn's post-test, respectively. The significance level applied was 95% (p=0.05).

## Results

### Clinical observations

There was no implant loss during the evaluated period, and all implants were included in the study. Implants from the control group (without ligatures) presented clinically healthy peri-implant soft tissue without bleeding or signs of inflammation. A few dental implants from the test group (dental plaque accumulation by cotton ligatures) showed exposure of threads and significant dental plaque accumulation.

### Histometric evaluation

**Figure 2:**
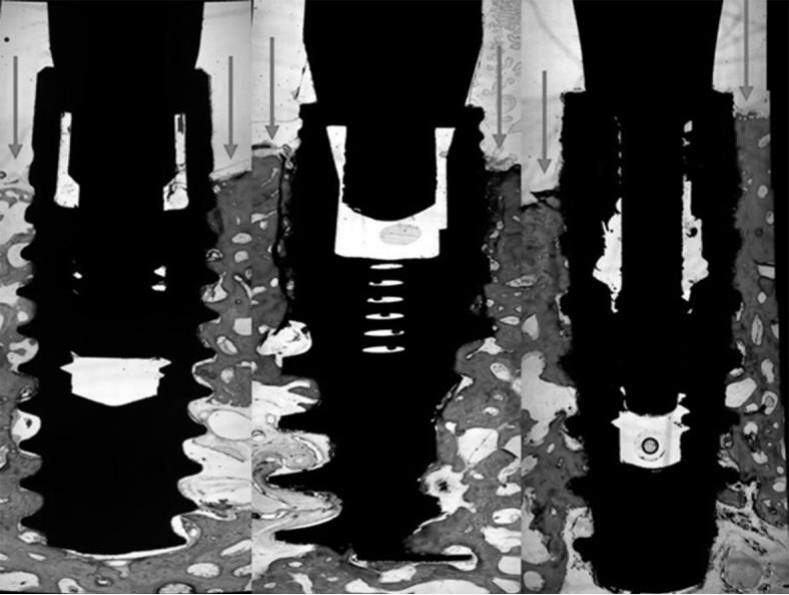
. Dental implants from the test site (with dental plaque accumulation and cotton ligatures). From left to right: Straumann, Nobel, and Plenum. The red arrows depicted the peri-implant bone loss in both buccal and lingual sites.

**Figure 3:**
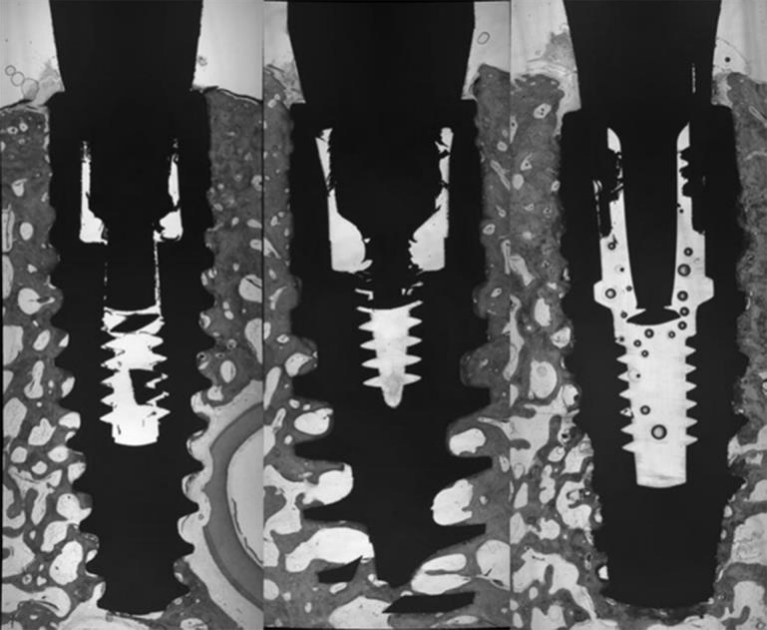
Dental implants from the control site (without ligatures). From left to right: Straumann, Nobel, and Plenum. Note that the peri-implant bone level surrounds the implant neck.

The peri-implant soft and hard tissues from dental implants from the control group generally appeared healthy, with the junctional epithelium extending a short distance apical to the implant shoulder. The newly formed bone was mostly lamellar and compact; numerous osteocytes were present in their lacunae. In some cases, osteoblasts were connected to the newly formed bone, indicating ongoing bone formation, and minor apposition of new bone could be found, specifically next to the thread area. Dental implants from the test group presented an apical migration of the junctional epithelium until the bottom of the peri-implant defect (Figures 2 and 3). 

**Figure 4:**
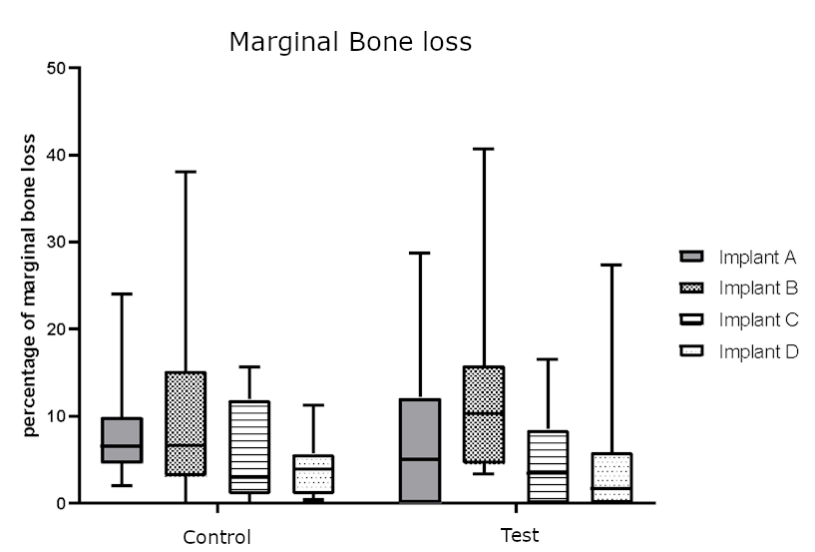
Box plots of the percentage of peri-implant bone loss from control (without ligature) and test (with ligature and dental plaque accumulation), p<0.05. Implants A, B, C, and D are Straumann, Nobel, Plenum, and Plenum submerged (negative control).

**Figure 5:**
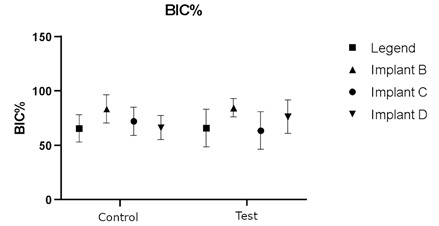
Dot plot of the percentage of BIC area alongside the implant from control (without ligature) and test (with ligature and dental plaque accumulation), p>0.05. Implants A, B, C, and D are Straumann, Nobel, Plenum, and Plenum submerged (negative control).

**Figure 6:**
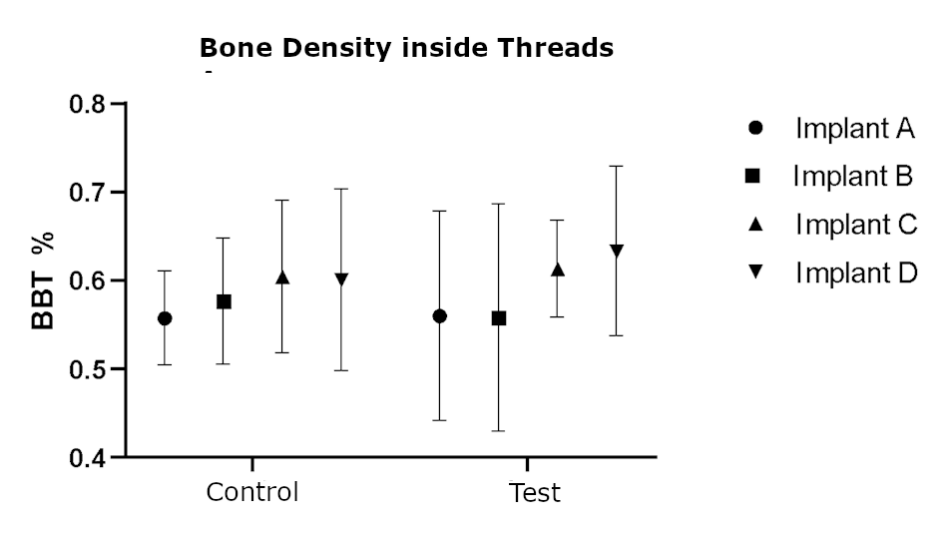
Dot plot of the percentage of peri-implant density inside the thread area (BBT) alongside the implant from control (without induced periimplantitis) and test (with induced periimplantitis), p>0.05. Implants A, B, C, and D are Straumann, Nobel, Plenum, and Plenum submerged (negative control).

Newly formed bone covered the specimens from submerged Plenum implants (negative control) over the implant head. Histometric variables for Marginal bone loss and Bone between threads (BBT) do not present statistical differences between groups (p>0,05). Bone to implant (BIC) analysis showed a statistical difference between groups C and B. (p=0.0152). The analysis of bone volume is presented in Figures 4, 5, 6, and Table 1.

**Table 1:** Bone to implant contact (BIC%), in implants with different manufacturing methods and surface treatments, placed into minipig mandibles with or without induced peri-implantitis. (Mean ± 95%CI).

Implant	Induced periimplantitis		TOTAL
	No	Yes	
A	65.45 ± 8.13aA	65.67 ± 10.72aA	65.56 ± 6.65
B	83.39 ± 11.07aA	84.29 ± 7.08bA	83.84 ± 6.38
C	72.01 ± 8.08aA	63.49 ± 10.79aA	67.75 ± 6.84
D	66.12 ± 7.66aA	76.24 ± 10.06abA	71.48 ± 6.71
TOTAL	71.91 ± 4.86	72.01 ± 5.46	

### MicroCT scan

**Figure 7:**
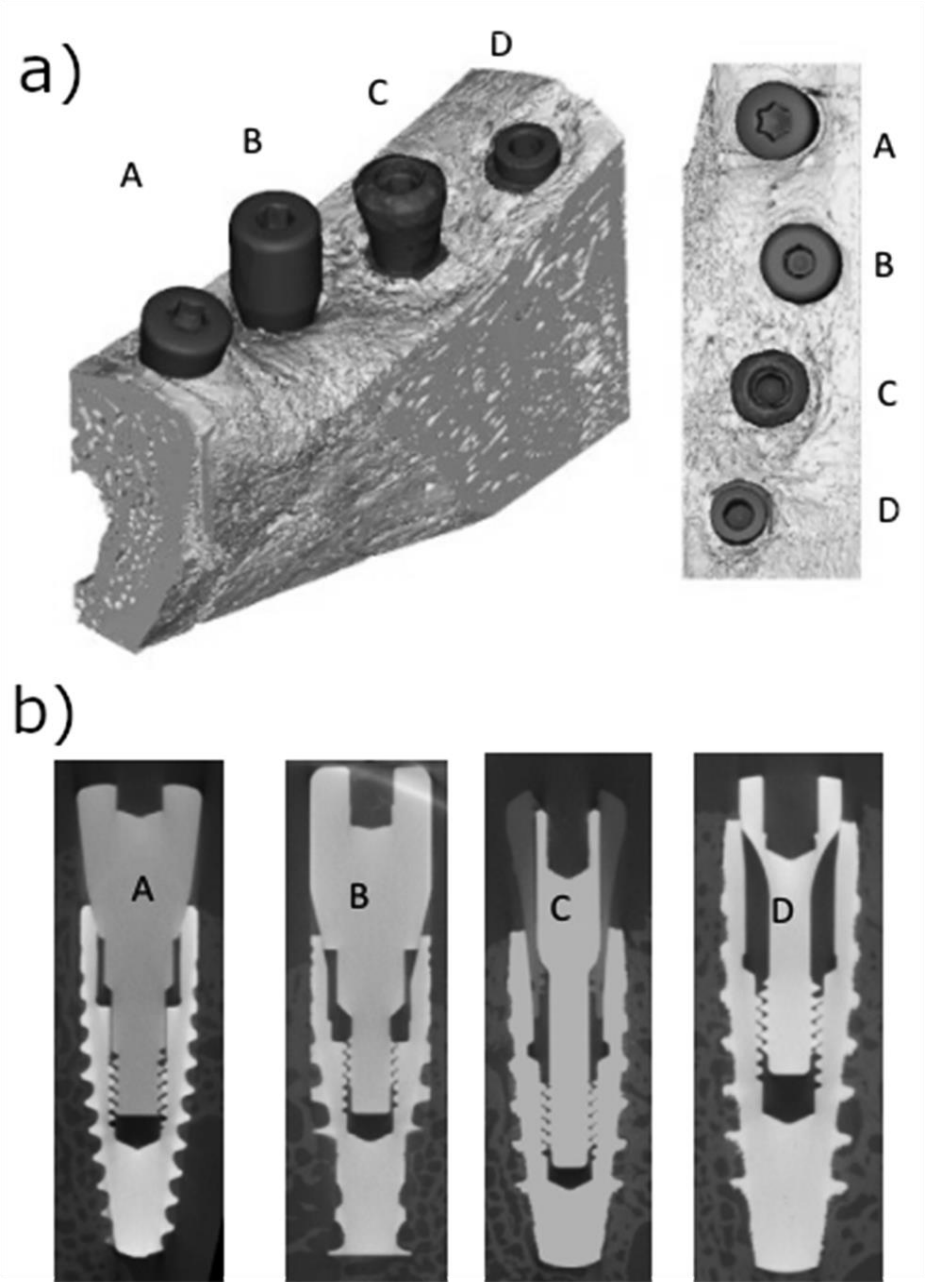
(A) Three-dimensional reconstruction (oblique and top view) of micro CT scans obtained at the test side, with induced periimplantitis, showing a similarity in the marginal bone loss pattern for the tested implant and commercially available brands. (B) Micro CT sample images of implants placed in minipigs' mandibles, with periimplantitis (test). Implants A, B, C, and D are Straumann, Nobel, Plenum, and Plenum submerged (negative control).

The scanned mandibles of minipigs were rendered into three-dimensional images for qualitative analysis. In the images it was possible to observe the shape and characteristics of marginal bone loss around the implants, with a similar pattern in the control group without induced periimplantitis, in the test group, all the implants presented a oblique bone loss all around the margin of implants, which is expected, the implants A and C presented marginal bone loss in periimplantitis similar to a cup, while B implants presented a straight and deep pattern of marginal bone loss. In both test and control, the Plenum implant presented a similar behavior to the commercially available brands, and none have shown a difference from the submerged implant (Figures 6 and 7).

## Discussion

Macro and microgeometry of dental implants play important roles in promoting osteoblast adhesion to the implant surface, but implant surfaces in a contaminated environment are susceptible to chemical and physical changes in their micro and nano structure, which could facilitate adhesion of bacteria and inflammatory cells^6^ ^,^ ^8^. Additive manufacturing allows higher control of the implant's topography, due to its reproducibility.^17^.

Implants fabricated by additive manufacturing have already shown promising results in studies evaluating cell adhesion to their surface. In an *in vivo* study, the topography of the additive-manufactured implants has presented a stronger and more substantial fibrin net than other surface treatments^18^. In line with *in vitro* findings, it has been demonstrated that additive manufacturing surfaces exhibit a distinct pattern of microbial colonization compared with machined titanium surfaces, suggesting that additive manufacturing may provide biological advantages regarding bacterial adhesion ^12^.

In our study, regarding the marginal bone loss at the cervical third of implants and crestal bone, the additive-manufactured implants presented similar results to the commercially available brands compared. The bone-to-implant contact (BIC) percentage was higher for implant B, but the test presented no statistical difference to implant A or to the negative controls. 

To contextualize these results, it is important to note that, in the literature, BIC is widely employed as a histomorphometric outcome to assess peri-implant bone healing and the quality of osseointegration achieved^7^. In the present study, a statistically significant difference was observed between groups B and C, whereas no significant difference was found between groups A and C. Additionally, the BBT parameter showed no statistically significant differences among any of the evaluated groups. These results indicate that, although the additive-manufactured implants differed significantly from group B in BIC, their overall osseointegration pattern, considering both BIC and BBT, remained consistent with that of the commercially available implants, suggesting a comparable biological performance in terms of bone healing and osseointegration^6^ ^,^ ^7^ ^,^ ^8^ ^,^ ^12^.

In this study, the microCT analysis allowed the visualization of distinct morphological patterns of marginal bone loss among the evaluated implants. While groups A and C exhibited a cup-shaped resorption profile, group B showed a straighter and deeper bone loss configuration.

Accordingly, marginal bone loss is considered the most reliable parameter to estimate the presence and progression of peri-implantitis, and its evaluation must account for changes beyond the expected physiological bone remodeling^4^ ^,^ ^6^. Although no statistically significant difference in marginal bone loss was observed between groups, qualitative microCT evaluation revealed distinct morphological patterns. Implants presenting vertically oriented defects are often associated with more advanced lesions and accelerated breakdown^19^, whereas shallower, cup-shaped configurations tend to indicate a more contained response and limited resorptive progression^20^. These findings are in line with experimental observations describing variations in defect morphology during the progression of peri-implantitis^7^ ^,^ ^11^, and further suggest that implants from groups A and C exhibited a more stable marginal bone profile, with morphologies indicative of a contained or less aggressive resorptive process. This trend, while not conclusive, may reflect surface-related or biological factors influencing local bone remodeling dynamics.

The inclusion of bilaterally submerged Plenum implants as negative controls enabled the evaluation of the intrinsic osseointegration potential of the additive manufacturing surface without the interference of microbial or inflammatory stimuli^8^ ^,^ ^12^ ^,^ ^17^. This experimental design allowed a more accurate distinction between the bone response to induced peri-implant inflammation and the baseline biocompatibility of the implant surface^4^ ^,^ ^7^ ^,^ ^11^, reinforcing that the biological behavior observed in the challenged experimental groups can be attributed primarily to the peri-implantitis induction process, rather than to any deficiency in the material’s osseointegration capacity or biocompatibility^7^ ^,^ ^8^.

Nevertheless, the literature highlights the challenge of balancing the gains in bone contact with the increased susceptibility to bacterial adhesion on rougher surfaces or those with nanotopography^4^ ^,^ ^6^ ^,^ ^7^. In this regard, although the available data remain limited, additive manufacturing appears promising by allowing a precise design control and microstructural standardization, which may potentially enhance the predictability and reproducibility of outcomes^7^ ^,^ ^8^ ^,^ ^9^ ^,^ ^17^, and could, in the future, enhance the development of decontamination protocols with nanometric spaces between implant pores and rugosities.

## Conclusion

Our results suggest that additive manufacturing implants have a similar behavior to commercially available implant brands with different surface treatments, appearing to be a valuable alternative to the fabrication of osseointegrated implants. Within the limits of this study, although not statistically significant, the results suggest that Straumann and Plenum showed a lower tendency toward peri-implant bone loss in this sample. 

## Data Availability

The research data are available within the article.
